# Wildfires and Public Health: A Comprehensive Review of Human‐Centric Studies

**DOI:** 10.1029/2025GH001534

**Published:** 2026-02-18

**Authors:** Xinyue Ye, Yuning Ye, Xiao Huang, Tracy Onega

**Affiliations:** ^1^ Department of Landscape Architecture & Urban Planning Texas A&M University College Station TX USA; ^2^ Department of Environmental Sciences Emory University Atlanta GA USA; ^3^ Department of Population Health Sciences University of Utah Salt Lake City UT USA

**Keywords:** wildfires, public health, human‐centric studies, systematic review

## Abstract

In the last decade, wildfires have surged in frequency, as highlighted in the 2024 National Interagency Fire Center report, and continue to rise, making them a worldwide concern due to their environmental and public health impact. Climate change and shifting fire patterns contribute to this growing challenge. This review addresses the complex relationship between wildfires and public health, facilitating informed decision‐making in response to this global challenge. Wildfires intricately affect human health, encompassing physical, psychological and social dimensions. Beyond immediate risks like respiratory issues, cardiovascular incidents, and burns, their enduring effects include prolonged exposure to poor air quality, population displacement, disrupted healthcare, psychological trauma and negative economic impacts. As research methods advance, it is vital to systematically review the existing literature to consolidate knowledge, identify gaps, and guide policies and interventions. Our review aims to provide a comprehensive overview of the health consequences linked to wildfires by synthesizing findings from diverse studies. We systematically reviewed 139 peer‐reviewed studies published between 1997 and 2023, retrieved from Web of Science, to synthesize evidence on wildfire exposure metrics, health impacts, and population vulnerabilities. We seek to outline the spectrum of health outcomes, explore potential impact mechanisms, and identify vulnerable populations. Additionally, we critically assess study methodologies, evaluate evidence quality, and pinpoint areas requiring further exploration.

## Plain Language Summary

Wildfires are happening more often and becoming more severe around the world, driven largely by climate change and changing fire behavior. According to the 2024 National Interagency Fire Center report, this trend is expected to continue, posing growing risks to both the environment and public health. This review looks at how wildfires affect human health across short‐ and long‐term timeframes. Immediate health concerns include breathing problems, heart conditions, burns, and injuries. Over time, wildfires can lead to lasting challenges such as poor air quality, displacement, mental health issues, disrupted healthcare access, and economic stress, especially for vulnerable groups. As wildfire risks grow, it's critical to understand their full impact on health to guide effective policies and responses. This review brings together findings from a wide range of studies to clarify what is known, highlight gaps in evidence, and support data‐driven planning. We assess how different studies measure health impacts, identify which populations are most at risk, and outline where further research is needed to strengthen interventions and protect communities.

## Key Points

- We conducted a systematic review of human‐centric studies examining the impacts of wildfire on public health
- We identified uneven research coverage across populations, study designs, and themes, especially in mental health and demographics
- We highlight the need to apply innovative machine‐learning to advance wildfire‐health research and enhance predictive capacity

## 1 Introduction

According to the most recent National Interagency Fire Center (NIFC) report (2024), the United States has experienced over 530,000 wildfire incidents in the past decade, burning more than 70 million acres (National Interagency Fire Center, 2025). Wildfires, natural disasters of increasing global concern, have garnered substantial attention due to their widespread occurrence, intensification, and profound influence on environmental and public health. With mounting evidence of climate change and altered fire regimes, wildfires have emerged as a critical challenge facing societies worldwide (J. C. Liu, Wilson, Mickley, Dominici, et al., 2017). Beyond their immediate destructive impact on ecosystems and property, wildfires significantly affect human health, causing a range of direct and indirect health outcomes that extend well beyond the confines of fire‐affected regions.

The health impacts of wildfires are complex and multifaceted, encompassing both physical and psychological dimensions. In recent decades, due to the escalation in the frequency and severity of wildfires, significant research attention to the potential consequences for human health is mounting. The interactions between combustion, particulate matter diffusion, and restricted geographic access, are multi‐faceted and poorly understood in the context of human health.

The existing systematic literature reviews about the health impact of wildfire range from the specific health impacts of wildfires, such as respiratory and cardiovascular issues, and immediate psychiatric implications like PTSD, anxiety, and depression (Foo et al., 2023; G. Liu et al., 2015; L. Zhang et al., 2023). While these studies provide valuable insights, there is a notable gap in understanding the comprehensive health effects of wildfire exposure. The existing reviews focus in single vulnerable populations such as children, the elderly, and those with pre‐existing health conditions instead of understanding the total population as a whole (Dittrich & McCallum, 2020; Gao et al., 2023; Henry et al., 2021; S. Zhang et al., 2023). Another critical area that appears underexplored is the existing literature reviews mostly focused on did not cover the research methods and their change in the articles. Furthermore, the health consequences of wildfires lack the broader and long‐term societal and economic repercussions that exacerbate health vulnerabilities (Jiao et al., 2023). This new systematic review will address these gaps by focusing on studies with different research methods that explore the prolonged health effects of wildfires, the indirect health consequences on diverse population groups, and the effectiveness of various public health interventions and policies aimed at mitigating these impacts. The review will also compare the situation of the wildfire all over the world and existing studies in health impacts, thereby providing a comprehensive understanding of the multifaceted health challenges posed by wildfires.

While the immediate health risks of wildfires are well‐documented—including respiratory ailments, cardiovascular incidents, and burns—emerging evidence suggests that their aftermath can cast a longer shadow on public health. The prolonged exposure to degraded air quality, the displacement of populations, the disruption of healthcare services, and the psychological trauma of witnessing and surviving wildfire events present additional layers of health vulnerability that warrant comprehensive investigation.

As the wildfire landscape evolves, so too does our understanding of their health impacts. Advances in research methodologies such as machine learning or deep learning, coupled with the availability of diverse data sets and analytical tools such as GIS and generative AI, have enabled researchers to explore the nuances of these complex relationships with increasing precision (Zhou et al., 2025). A systematic synthesis of existing literature on the health impacts of wildfires is essential to distil current knowledge, identify gaps, and inform policy, preparedness, and public health interventions.

**Figure 1:**
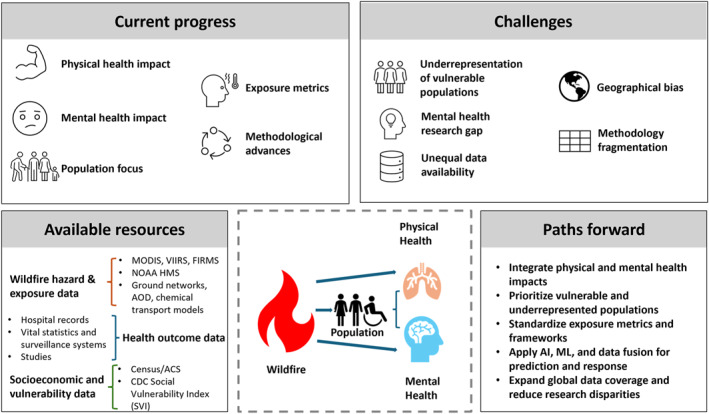
The structure of this review.

In the subsequent sections of this review, we will delve into specific health dimensions influenced by wildfires, including respiratory health, cardiovascular health, mental well‐being, and the social demographic information. Additionally, we will discuss the methodological challenges and opportunities encountered in wildfire health impact research. We would like to contribute to a deeper understanding of the health implications of wildfires and foster a more resilient and informed response to these increasingly prevalent natural disasters. The structure of this review is presented in Figure 1.

## 2 Methodology

### 2.1 Study Eligibility Criteria

To better capture the diversity of wildfire exposure metrics, we categorized studies into three primary exposure types: (a) burned area or proximity‐based measures, (b) air quality and smoke‐related indicators (e.g., PM2.5 concentrations), and (c) satellite‐detected fire events. This categorization enabled clearer comparisons across study designs and outcomes and allowed us to explore how different exposure measures influence reported health impacts. First, we selected studies focused on public health and excluded those centered on chemical materials, biological processes of wildfires, or topics not significantly relevant to public health. Second, we included studies focused on human health, excluding those focused on animals or botanical landscapes. Third, we included studies specifically addressing wildfires, excluding those on broader climate change or other types of fires (e.g., coal‐mine fires, fuel‐related fires). Fourth, we excluded studies focused on groups distinct from the public (e.g., firefighters and emergency workers).

We classified each study into one of the following epidemiologic designs: retrospective cohort, prospective cohort, case‐crossover, time‐series, cross‐sectional, or ecological. This taxonomy follows conventions in prior reviews of wildfire smoke health impacts (Reid et al., 2015). When a study superficially fits multiple categories, we applied a hierarchical rule based on the primary analytical estimator and causal contrast (priority: cohort → case‐crossover → time‐series → cross‐sectional → ecological). This procedure reduces overlap and ensures consistency in classification across diverse studies.

### 2.2 Search Strategy

We conducted a comprehensive literature search in September 2023 using Web of Science, the database most relevant to our review. The search employed three groups of keywords, each related to specific domains: (a) Wildfires, including terms such as “wildfire*,” “forest fire*,” and “bushfire*”; (b) Health, incorporating keywords like “health,” “well being,” and “well‐being”; and (c) Human‐centric studies, including terms such as “human,” “people,” and “individual.” Two criteria were applied to all database searches: (a) the studies had to be written in English, and (b) they needed to be published as full articles in peer‐reviewed journals or conference proceedings. In addition to database searches, we reviewed references and citations of the eligible studies to identify additional relevant records.

### 2.3 Screening Process

**Figure 2:**
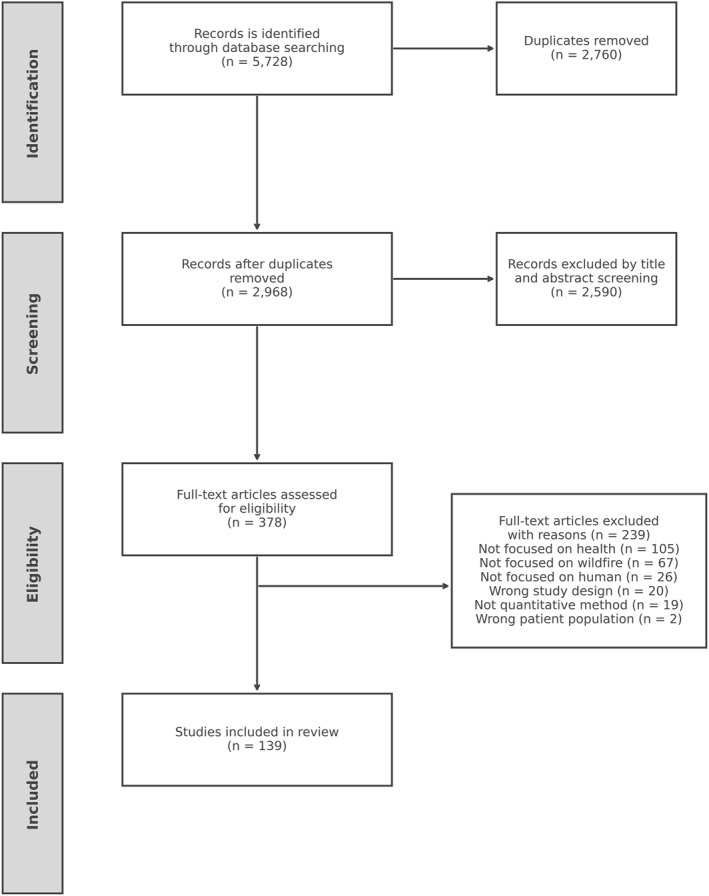
PRISMA flow diagram for this review study.

Figure 2 outlines the article screening and identification process, adhering to the guidelines specified in the Preferred Reporting Items for Systematic Reviews and Meta‐Analyses (PRISMA) (Moher et al., 2009). After removing duplicates, 2,968 records were identified for further eligibility screening (see Figure 1). During title and abstract screening, 2,590 articles were excluded based on the following criteria: (a) studies not addressing human health outcomes (e.g., focusing solely on ecological or animal impacts); (b) non‐wildfire fire events such as industrial or indoor fires; (c) reviews, commentaries, or modeling papers without original data; and (d) mechanistic or laboratory studies not directly connected to public health impacts. These criteria ensured that the included studies directly addressed human health outcomes associated with wildfire exposure. Full texts were then assessed against predefined inclusion criteria, which required (a) peer‐reviewed status, (b) focus on wildfire exposure as a primary variable, and (c) original empirical analysis. Studies were excluded if they focused solely on ecological impacts, modeled fire behavior without health outcomes, or lacked sufficient methodological detail. Discrepancies in screening decisions were resolved through discussion until consensus was reached. Based on this, an additional 239 articles were excluded during the full‐text assessment stage. Ultimately, 139 studies met the inclusion criteria and were included in this review.

### 2.4 Data Extraction

Data were extracted from each selected study across five principal dimensions: study design, data collection methods, wildfire location, health impacts, and conclusions. Detailed characteristics extracted for analysis included the first author, publication year, study design, study period, duration of intervention, study location, study setting, data source, key findings, health impact categories, wildfire types, and population demographics. Data extraction was systematically performed using a custom online form developed via Google Forms to ensure consistency and accuracy in gathering information. For the burned area and number of fires for the countries, we retrieve the data set from the Global Wildfire Information System (GWIS) (Artés et al., 2019).

## 3 Results

### 3.1 Characteristics of the Reviewed Studies

#### 3.1.1 Increasing Numbers of Publications in Recent Years

**Figure 3:**
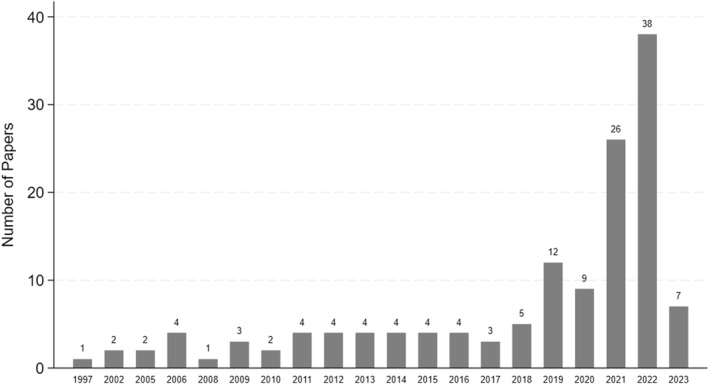
The frequency of selected studies by publication year.

In the late 1990s and early 2000s, scholarly publications on the health impacts of wildfires were sparse, with a notable article appearing in 1997 followed by a hiatus until 2002 (Figure 3). The recognition of wildfires' health ramifications gradually heightened, necessitating increased research attention. This need was compounded by the rising frequency and severity of wildfires across regions such as California and Australia, further spotlighted by climate change‐related phenomena including prolonged droughts and extreme weather events. These environmental changes have led to more frequent wildfires, thereby escalating research into their health implications, particularly concerning respiratory health due to smoke and deteriorating air quality. By 2002, there was a discernible increase in the volume of research, which surged significantly from the mid‐2010s onwards. The recurrent, devastating wildfires in areas like the Western United States and Australia likely spurred this surge, along with heightened public and policy focus on wildfire management and health impacts. Moreover, advancements in data collection methods, including enhanced remote sensing and air quality monitoring techniques, have substantially supported these research endeavors. Consequently, the years 2021 and 2022 marked a peak in publications, reflecting the urgent ongoing need for understanding and mitigating the health effects of wildfires.

#### 3.1.2 Unstable Occurrence of Wildfire in the World

**Figure 4:**
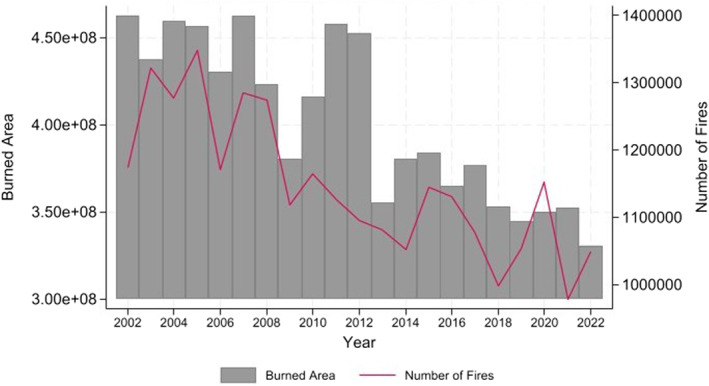
Yearly burned area and yearly number of fires in the world (European Commission, Joint Research Centre (JRC, 2024)).

Figure 4 presents data on global wildfire activity from 2002 to 2022. The chart illustrates a fluctuating yet generally decreasing trend in the yearly burned area. Meanwhile, the number of fires shows a pronounced drop in the first decade. In the second decade, the data indicates variability in the number of fires, oscillating between 650,000 and 750,000, with an overall increasing trend toward the latter years. This variability highlights the unpredictable nature of wildfire occurrences and the complex interplay of factors such as climatic conditions, fire management practices, and ecological changes that affect fire behavior and impact.

To provide a more meaningful context beyond simple event counts, we also examined severity‐related indicators, including total burned area per event and estimated population exposure in affected regions. This approach highlights that the societal and research impacts of wildfires vary widely: smaller fires in remote areas often generate limited academic attention, whereas large‐scale, high‐impact events near densely populated urban centers (e.g., the 2018 Camp Fire in California) trigger extensive research activity. Including these severity and exposure metrics enables a more nuanced understanding of how wildfire magnitude and community vulnerability relate to research output.

#### 3.1.3 Countries of the Publication

**Figure 5:**
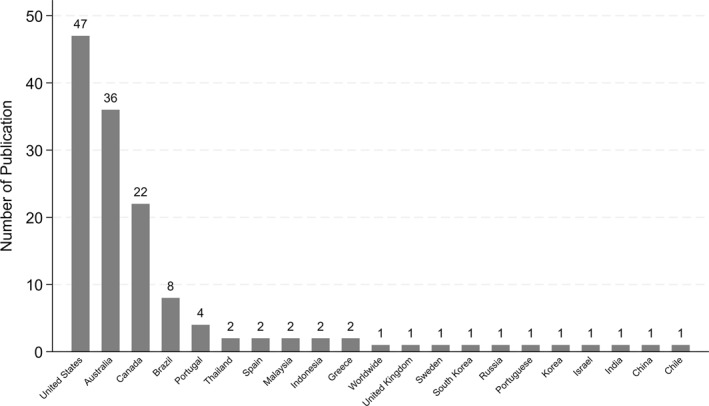
Number of publications by country of wildfire location.

Figure 5 delineates the distribution of scholarly publications related to wildfires across various countries. It clearly shows that the United States leads with a total of 47 publications, followed closely by Australia with 36, and Canada with 22. Brazil and Portugal are also notable contributors with 8 and 4 publications, respectively. The rest of the countries, including Thailand, Spain, Malaysia, Indonesia, Greece, Worldwide, United Kingdom, Sweden, Saudi Arabia, Russia, Portugal, Korea, Israel, India, China, and Chile, each report fewer publications, ranging from one to two. This disparity underscores a significant concentration of wildfire research in a select few countries, with the United States, Australia, and Canada emerging as the predominant centers of academic output in this field. This trend may reflect the higher incidence of wildfires in these regions or possibly more substantial research funding and resources dedicated to understanding and mitigating wildfire impacts.

### 3.2 Results in Physical Health

#### 3.2.1 Characteristics of Publication in Physical Health

**Figure 6:**
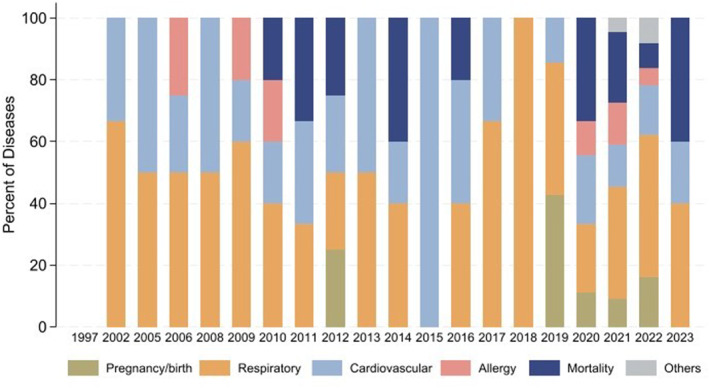
Categories of physical disease by year.

Figure 6 provides a detailed overview of the categories of physical diseases by year in terms of their percentage distributions. The most prominent category throughout the years has been respiratory diseases, consistently accounting for a significant portion annually, underscoring a persistent public health challenge. Cardiovascular problems remain stable, fluctuating slightly each year. Allergy instances also exhibit minor variations, generally occupying a smaller percentage. However, the years 2021 and 2023 observed a sudden spike in mortality rates, suggesting the potential impact of COVID‐19 during these years. Other physical diseases displayed a notable presence throughout the years, with a particularly high percentage in 2023 meanwhile. This evolving health landscape over the past two decades is indicative of changing health challenges. Future studies should prioritize investigating the factors behind the escalated mortality rates and the notable presence of other diseases to devise responsive healthcare strategies. Because the number of included studies is limited, some years contain only one or very few publications, which may introduce bias into the observed distribution. Future reviews incorporating a larger body of studies will be needed to more accurately capture year‐to‐year trends and shifts in publication focus.

#### 3.2.2 Summary of Findings in Physical Health

First, intense bushfire smoke exposure results in both acute and persistent symptoms among people with severe asthma (Beyene et al., 2022). The wildfire also increased on the hospital admissions as they are highly linked to PM10 and black carbon exposure, with strong effects from fine particulate matter (PM2.5) (Cleland et al., 2021; Crabbe, 2012; Wen et al., 2022), particularly the pregnant woman with asthma and Indigenous populations (Beyene et al., 2022; Hanigan et al., 2008; Murphy et al., 2022). In addition, wildfire‐specific PM2.5 is associated with salbutamol dispensations (Elliott et al., 2013) and increased odds of respiratory physician visits and hospital admissions (Henderson et al., 2011). What's more, there are also increased respiratory‐related hospital visits directly due to wildfire smoke (Casey et al., 2021; DeFlorio‐Barker et al., 2019), which also increased risk of developing respiratory illness in children (Abdo et al., 2019; Barbosa et al., 2024; Ciciretti et al., 2022). Higher risk of outpatient visits for respiratory conditions, with significant interactions between smoke event days and high temperatures (Heaney et al., 2022; Mahsin et al., 2022), which could also be caused by wildfires. Long‐term exposure to wildfire smog is also associated with obstructive lung abnormalities (Ontawong et al., 2020).

Second, the cardiovascular admissions are also strongly associated with same‐day PM exposure, especially fine particulate matter (PM2.5) (Crabbe, 2012). PM2.5 exposure is linked to increased out‐of‐hospital cardiac arrests and ischemic heart disease (Haikerwal et al., 2015). Increased cardiovascular hospital admissions with wildfire PM2.5 exposure, particularly affecting the elderly (Cleland et al., 2021; Mahsin et al., 2022).

Third, wildfire‐related PM2.5 is associated with increased mortality from various cancers (Yu et al., 2022). Specifically, increased incidences of lung cancer and brain tumors have been noted among populations exposed to wildfire smoke (Korsiak et al., 2022). The studies also revealed that exposure to wildfire‐related PM2.5 concentration was linked to increased mortality from nasopharynx, oesophagus, stomach, colon/rectum, larynx, skin, breast, prostate, and testis cancers (Yu et al., 2022).

Last but not least, PM2.5 exposure during wildfires was also associated with increased outpatient visits for conditions such as congestive heart failure and ischemic heart disease among seniors (Mahsin et al., 2022). Besides, increased odds of dispatches related to diabetic conditions during wildfire seasons were observed, suggesting that PM2.5 exposure impacts chronic conditions like diabetes (Yao et al., 2020). For the long‐term impacts, exposure to wildfire smoke has potential long‐term adverse health effects, including changes in lung function parameters indicative of chronic respiratory issues (Orr et al., 2020). Chronic respiratory diseases, such as chronic obstructive pulmonary disease (COPD), were also found to be exacerbated by wildfire smoke, especially in older populations (Magzamen et al., 2021; Morgan et al., 2010). Moreover, exposure to wildfire smoke has been associated with increased usage of medications for respiratory conditions and chronic diseases, suggesting a higher burden of disease management during and after wildfire events (Künzli et al., 2006).

### 3.3 Results in Mental Health

#### 3.3.1 Characteristics of Publication in Physical Health

**Figure 7:**
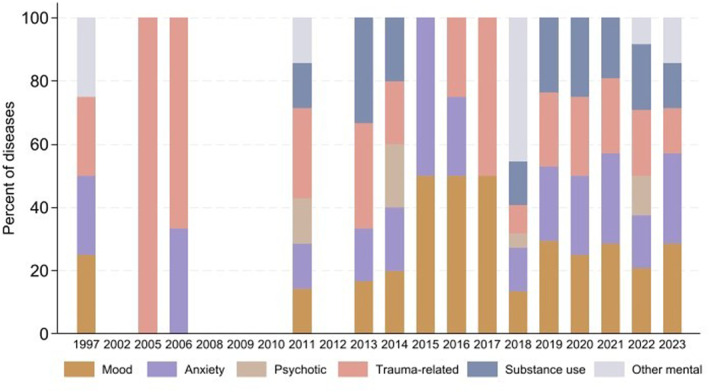
Categories of mental diseases by year.

Figure 7 reveals that mood disorders have been a consistent and significant category, highlighting an ongoing mental health concern. Anxiety disorders also show a stable presence across the years, indicating their persistent impact on public mental health. Psychotic disorders exhibit noticeable fluctuations but remain a significant category. Trauma‐related disorders and substance use disorders display variability over the years, with some periods showing a higher prevalence. Notably, the years 2021 and 2023 observed an increase in other mental disorders, suggesting the emergence or increased recognition of other mental disorders during these years. This evolving mental health landscape over the past decades reflects changing patterns and possibly the influence of external factors such as societal changes and global events. Future studies should prioritize understanding the underlying causes of these trends and the factors contributing to the observed fluctuations to develop effective mental health strategies and interventions.

#### 3.3.2 Summary of Findings in Mental Health

First, wildfires have a profound and enduring impact on mood disorders, with depression being a significant concern. Research shows that communities affected by bushfires continue to experience high levels of psychological distress and depression for up to a decade after the event (Bryant et al., 2021). Persistent PTSD and depression are common, highlighting that mood disorders remain a major issue long after the fires (Bryant et al., 2014). Individuals with a history of depression or those who experience unemployment are particularly vulnerable to developing Major Depressive Disorder (MDD) following wildfires (Mao et al., 2022). Young adults also face increased risks, with significant rises in depressive symptoms observed after wildfire events (Parslow et al., 2006). For instance, students at Keyano College reported heightened mental health issues, including depression, following wildfire exposure (Ritchie et al., 2021). Lower resilience among youth has been linked to worse mental health outcomes, including increased depression, in the aftermath of such disasters (Brown et al., 2019a).

Second, anxiety disorder is another major concern following wildfire exposure. Keyano College students showed increased levels of anxiety disorders post‐wildfire (Ritchie et al., 2021), a trend that extends to the general population. Residents seeking care from out‐of‐hours primary care clinics also reported heightened anxiety levels after wildfires (Moosavi et al., 2019). Even a year after the fires, evacuees continued to experience significant anxiety symptoms, indicating a long‐lasting impact (Belleville et al., 2021). Mindfulness practices have been identified as helpful in mitigating anxiety, suggesting potential strategies for managing mental health in the wake of such events (Silveira et al., 2021).

Third, trauma‐related symptoms, particularly PTSD, are prevalent among those affected by wildfires. The risk of PTSD increases with factors such as social fragmentation, property damage, and perceived community cohesion (Bryant et al., 2017). Symptoms of PTSD, including flashbacks and intrusive thoughts, can persist long after the disaster (Bryant et al., 2018; Moosavi et al., 2019; Usher et al., 2022). Young adults, in particular, show a notable increase in PTSD symptoms following bushfires (Parslow et al., 2006). Even a year after the fires, evacuees continue to report significant PTSD symptoms, illustrating the long‐term psychological impact of these events (Belleville et al., 2021; Hong et al., 2022).

Fourthly, Substance use disorders also see a notable rise following wildfire events. Students at Keyano College, for example, reported increased rates of substance use and alcohol‐related issues post‐wildfire (Ritchie et al., 2021). This trend is reflected in the general population, with higher prevalence rates of substance use disorders observed among residents seeking out‐of‐hours medical care (Moosavi et al., 2019). The persistence of substance use issues among evacuees a year after the fires further underscores the need for targeted interventions to address these challenges (Belleville et al., 2021).

Last but not least, in addition to mood disorders, anxiety, and trauma‐related symptoms, wildfire‐affected individuals often face a range of other psychological challenges. Persistent issues such as insomnia and increased stress are common among evacuees even a year after the fires (Belleville et al., 2021). Effective coping strategies, including social support and accurate information sharing, are crucial for maintaining mental health post‐disaster (Cavanagh et al., 2018; Gallagher et al., 2019; Verstraeten et al., 2021). Increased healthcare use for psychological symptoms highlights the broad range of mental health impacts that require comprehensive support and intervention (Brown et al., 2021; Moosavi et al., 2019).

### 3.4 Characteristics of Publications in Wildfire

**Figure 8:**
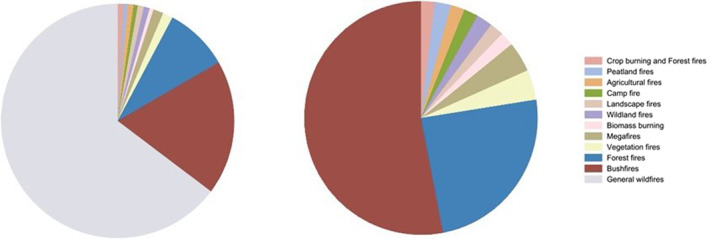
Categories of wildfires (with and without general wildfires).

In the first chart of Figure 8, general wildfires dominate the distribution, making up a significant portion of the pie. This indicates that a range of wildfires are categorized under this broad term. The other categories represent much smaller portions of the total, suggesting they are less frequent or less broadly categorized.

While the second chart excludes general wildfires and offers a more detailed look at the specific types of wildfires. Forest fires and bushfires become the most prominent categories, indicating that these are the most common specific types of wildfires when general wildfires are excluded. The remaining categories only represent minor portions of the pie.

### 3.5 Results of Publications in Population

#### 3.5.1 Characteristics of Publication in Population

**Figure 9:**
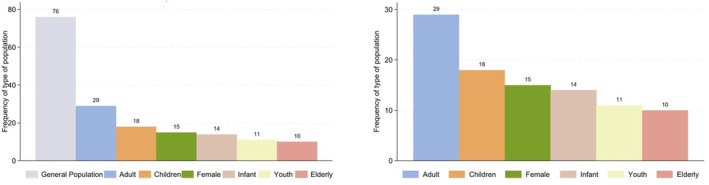
Distribution of population in publications (include and exclude the general population).

In the first chart of Figure 9, the category of general population is the most frequently studied, indicating a broad focus on studies that encompass a wide range of individuals without specific demographic distinctions. The next most frequently studied group is the adults with 29 publications, followed by the children at 18. There are 15 publications focus on the female, while 14 focusing on the children. The youth and the elderly are less frequently studied, with 11 and 10 instances, respectively.

While the second chart excludes the general population and reveals a more detailed focus on specific demographic groups. These figures highlight that certain vulnerable populations such as children, infants, youth, and the elderly are less frequently represented in publications compared to adults and the general population. This underrepresentation of vulnerable groups suggests a potential gap in research that could address specific health and social challenges faced by these populations. Children and infants, for instance, have unique healthcare needs and developmental considerations that require targeted research. Similarly, the elderly are often at higher risk for chronic diseases and other health issues, necessitating focused studies.

#### 3.5.2 Summary of Findings in Population

First, the health impacts of wildfire smoke exposure on women, particularly those with asthma, are profound. Prolonged exposure significantly affects the health of women with asthma, including pregnant women, who are at higher risk despite efforts to minimize exposure (Beyene et al., 2022; Murphy et al., 2022). Studies have shown that women and men experience and cope with bushfire impacts differently, with women more likely to use emotion‐focused strategies (Cavanagh et al., 2018; Rodney et al., 2021). Additionally, the risks of respiratory admissions due to wildfire smoke are significantly higher for women compared to men (J. C. Liu, Wilson, Mickley, Dominici, et al., 2017; J. C. Liu, Wilson, Mickley, Ebisu, et al., 2017). Adverse birth outcomes, such as preterm birth and lower birth weight, have been associated with maternal exposure to wildfire smoke during pregnancy (Abdo et al., 2019; Heft‐Neal et al., 2022; Holstius et al., 2012; Rangel & Vogl, 2019; Requia, Amini, et al., 2022; Requia, Kill, et al., 2022).

Second, Infants are particularly vulnerable to the adverse health effects of wildfire smoke exposure. Maternal exposure to smoke during pregnancy is linked to various adverse birth outcomes, including lower birth weight, preterm birth, and gestational diabetes (Abdo et al., 2019; Heft‐Neal et al., 2022; Holstius et al., 2012; Rangel & Vogl, 2019; Requia, Amini, et al., 2022; Requia, Kill, et al., 2022). Short‐term wildfire PM2.5 exposure can affect small airway function in infants, potentially leading to long‐term respiratory issues (Moitra et al., 2021). Increased respiratory morbidity among infants has been observed, highlighting the significant health risks posed by wildfire smoke (Santos et al., 2015; Schöllnberger et al., 2002). Furthermore, exposure to wildfires has been associated with an increased risk of congenital anomalies such as gastroschisis if mothers live close to the fires (Park et al., 2022).

Third, the elderly population is particularly susceptible to the health impacts of wildfire smoke exposure. Increased cardiovascular hospital admissions have been observed among seniors, particularly those exposed to PM2.5 from wildfires (Cleland et al., 2021; Mahsin et al., 2022). Higher risks of physician visits for congestive heart failure and ischemic heart disease among seniors in the post‐wildfire period have also been reported (Mahsin et al., 2022). Rapid increases in PM2.5 concentrations resulting from wildfire smoke can significantly impact the health of elderly populations, even those thousands of kilometers away from the fires (Le et al., 2014). Short‐term exposure to wildfire‐specific PM2.5 is associated with a higher risk of respiratory diseases in the elderly (J. C. Liu, Wilson, Mickley, Dominici, et al., 2017; J. C. Liu, Wilson, Mickley, Ebisu, et al., 2017). Additionally, the elderly face increased morbidity and mortality from cardiopulmonary issues during wildfire events (Magzamen et al., 2021).

Fourthly, children are particularly vulnerable to the adverse health effects of wildfire smoke. Increased hospital admissions for respiratory diseases, particularly among Indigenous children, have been documented (Hanigan et al., 2008). Children exposed to wildfire smoke are at a higher risk of developing respiratory illnesses, with studies showing that PM2.5 from wildfires has a greater impact on pediatric respiratory health compared to other sources (Abdo et al., 2019; Ciciretti et al., 2022). Long‐term exposure to wildfire smog has been linked to obstructive lung abnormalities in children, highlighting the need for effective preventive measures (Ontawong et al., 2020). Moreover, short‐term exposure to wildfire‐specific PM2.5 is associated with respiratory disease risks among children, with asthma exacerbation being a significant concern (Barbosa et al., 2022; Hutchinson et al., 2018; J. C. Liu, Wilson, Mickley, Dominici, et al., 2017; J. C. Liu, Wilson, Mickley, Ebisu, et al., 2017).

Finally, the mental health impacts of wildfires on youth are significant and long‐lasting. Adolescents are particularly vulnerable, with increased reports of depression and suicidal ideation following wildfire events (Brown et al., 2019b). Long‐term mental health impacts in youth and communities affected by wildfires underscore the need for multi‐year funding and support programs to address these issues (Brown et al., 2019a, 2021). Keyano College students, for example, reported higher levels of mental health issues, including anxiety, depression, and substance use, following wildfire exposure (Ritchie et al., 2021). The notable increase in PTSD symptoms among young adults post‐bushfire further highlights the psychological toll of these disasters (Parslow et al., 2006). Additionally, lower resilience among youth is associated with substantially worse mental health outcomes in the aftermath of such traumatic events (Brown et al., 2019a). Increased healthcare use for asthma and other respiratory conditions among children aged 0–5 due to wildfire smoke further illustrates the health risks faced by young populations (Heaney et al., 2022).

### 3.6 Characteristics of Publications in Methodology

**Figure 10:**
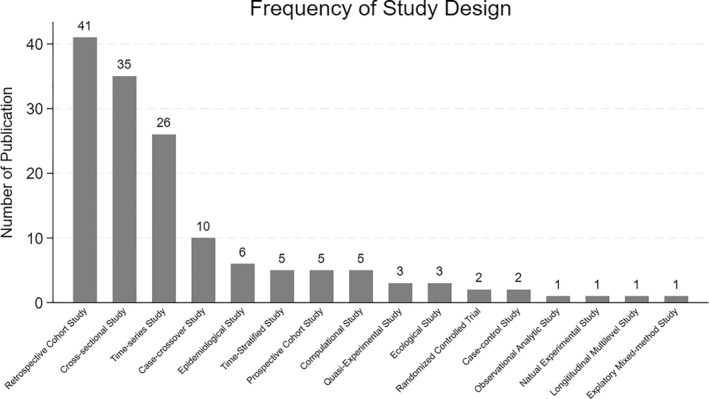
Frequency of study design.

Consistent with prior syntheses of wildfire and health literature (Adetona et al., 2016; Gould & Lewis, 2016; Kondo et al., 2018; Reid et al., 2015), studies clustered into two main groups based on design. The first focuses on short‐term acute exposure effects, primarily using time‐series and case‐crossover designs to examine immediate respiratory or cardiovascular outcomes. The second explores longer‐term or population‐level impacts using cohort or cross‐sectional approaches. This classification aids interpretation of causal strength and evidence maturity. Moreover, an increasing number of recent studies employ spatial modeling and machine‐learning techniques for exposure assessment, reflecting an evolving methodological frontier in wildfire epidemiology (Jain et al., 2020). Figure 10 presents a comparison of various research methodologies based on their frequency of use. Retrospective cohort studies are the most common, with 41 occurrences, indicating a strong preference or suitability for this study design in the given context. Cross‐sectional studies follow closely with 35 instances, while time‐series studies appear 26 times. Case‐crossover studies and epidemiological studies show moderate usage with 10 and 6 occurrences, respectively. It also illustrates a descending frequency with other study designs such as time‐series analysis, prospective cohort, combination, and quasi‐experimental studies each having 5 occurrences. Ecological studies and randomized controlled trials appear less frequently, with 3 and 2 instances respectively. The least common designs include case‐control, observational analytic, natural experimental, longitudinal analytic, and exploratory mixed‐method studies, each with only 1 occurrence.

This distribution highlights a pattern where observational study designs, such as retrospective cohort and cross‐sectional studies, are more frequently employed compared to experimental designs like randomized controlled trials. This preference may reflect the context's research constraints or objectives, favoring designs that allow for broader data collection and analysis over time or across populations.

**Figure 11:**
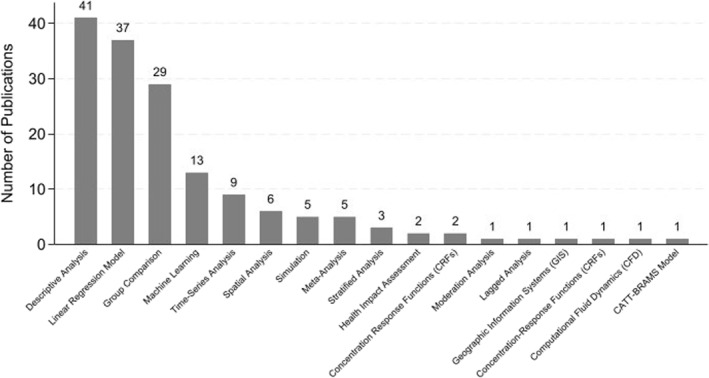
Frequency of methods.

Figure 11 illustrates the relative utilization of various statistical methods. Descriptive analysis emerges as the most frequent technique, with over 40 instances of application, underscoring its fundamental role in data interpretation. Linear regression model methods trail closely behind, indicative of their importance in contrasting different data sets. Group comparisons are also prominently utilized, likely reflecting their utility in outcomes modeling where the response variable is categorical. Machine learning shows moderate usage, suggesting their selective application in scenarios requiring prediction of continuous outcomes or complex pattern recognition. Spatial analysis, time‐series analysis and simulation are less frequently employed, pointing to their niche application in specific research contexts. This figure further demonstrates a minimal employment of advanced or specialized techniques such as stratified analysis and meta‐analysis, denoting their targeted use in studies where specific data structures are analyzed.

## 4 Discussion

### 4.1 Attention Drawing: Uneven Distribution of Burned Areas and Research in the World

In many parts of Central Africa, such as the Democratic Republic of the Congo (DRC) and Angola, traditional agricultural burning practices—closely tied to seasonal cycles and landscape conditions—contribute to the high frequency of fires. Angola's semi‐arid savanna climate, for instance, facilitate widespread burning, yet these events attract far less scholarly attention than high‐profile fires in regions such as the Amazon. This discrepancy persists even though the Congo Basin, often called the planet's second “green lung” after the Amazon, experiences frequent fires with significant ecological and social consequences (Vizzuality, 2024).

Importantly, while burned area provides a useful proxy for wildfire severity, it does not fully capture population exposure, health consequences, or infrastructural damage—dimensions that often drive the most serious community‐level impacts. Future research could integrate additional metrics such as smoke‐related population exposure, hospital admissions, or socioeconomic losses to better capture the multifaceted impacts of wildfires.

Beyond ecological conditions, disparities in research output are also shaped by deep structural inequities. Countries with robust monitoring systems, long‐term health data sets, and established research institutions—such as the United States, Australia, and Canada—tend to generate more wildfire–health studies even when their burned areas are comparable to those in regions with far fewer resources (Bowman et al., 2017; Johnston et al., 2012). Conversely, many low‐ and middle‐income countries experience large‐scale burning but lack the technical capacity, funding, or public health surveillance systems required to conduct wildfire epidemiology (Ford et al., 2017). As a result, the global body of literature increasingly reflects not only where wildfires occur, but where research infrastructure exists to analyze them.

Moreover, local fire regimes vary widely across ecological and cultural contexts. In parts of Africa, Southeast Asia, and South America, fire use is often tied to agricultural cycles, land‐clearing practices, or traditional ecological knowledge (Archibald, 2016; van der Werf et al., 2017). Differences in governance, land‐tenure systems, disaster reporting, and media coverage further influence whether wildfire events receive scientific attention (The Human Cost of Disasters, 2020). Collectively, these factors contribute to international disparities in how wildfire impacts are documented, understood, and addressed.

### 4.2 Addressing the Underexplored Terrain: Mental Health Research in the Wake of Wildfires

In recent years, the escalating impact of climate change has not only intensified environmental disasters but also heightened the necessity to focus on mental health outcomes that follow such events. of understanding their psychological consequences. Wildfires, as one of the most destructive climate‐related hazards, exert profound effects on mental health that extend far beyond the immediate physical damage. Exposure to wildfire events is strongly associated with acute psychological distress, including heightened levels of post‐traumatic stress disorder (PTSD), anxiety, and acute stress responses. These effects can stem from the immediate threat to life, loss of property, and the intense sensory experiences of evacuation and fire exposure (To et al., 2021). Children, in particular, may exhibit behavioral changes and heightened anxiety, while adults often report fear of recurrence, hypervigilance, and disrupted daily functioning in the aftermath of wildfire disasters.

Beyond these acute effects, wildfires also contribute to long‐term and indirect mental health consequences. Displacement, economic insecurity, disruption of social networks, and the cumulative stress associated with repeated exposure to fire events can lead to chronic depression, substance use disorders, and prolonged psychological distress (Friesinger et al., 2019). These findings underscore the importance of integrating mental health considerations into disaster response and recovery planning, ensuring that psychosocial support services are available both immediately following a fire and throughout the long‐term recovery process.

Despite the acknowledged link between wildfires and mental health outcomes, there remains a conspicuous gap in research dedicated to exploring this domain as we found that only 25% of the research paid attention to mental health and it has a trend of increase in recent years. Most existing research focuses on short‐term psychological impacts, while longitudinal studies on chronic outcomes and comparative analyses across demographic groups remain scarce. Vulnerable populations—including children, the elderly, Indigenous communities, and those with pre‐existing psychiatric conditions—are particularly understudied, despite evidence of heightened risk. Bridging this gap requires a concerted effort from the research community, policymakers, and mental health professionals to ensure that mental health considerations are integral to wildfire response and recovery strategies.

### 4.3 Exposure Metrics and Methodological Considerations

**Figure 12:**
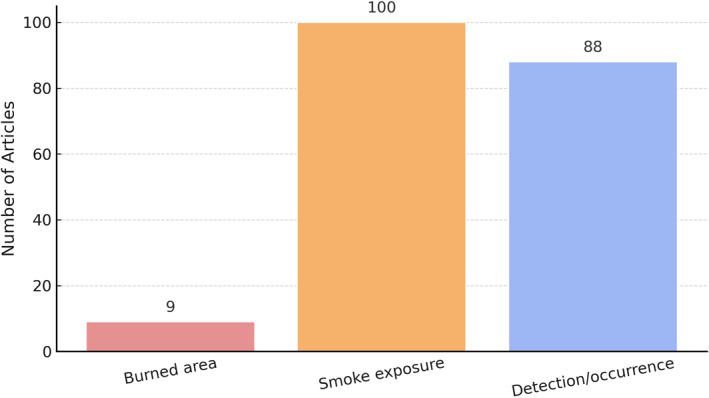
Distribution of wildfire exposure categories in publications.

The Figure 12 illustrates the frequency of three primary wildfire exposure metrics used across the reviewed literature: burned area (light red), smoke exposure (soft orange), and detection/occurrence (light blue). The predominance of smoke exposure reflects a strong focus on population‐level health impacts, while detection‐based and burned‐area measures are less frequently applied.

A key methodological observation emerging from this review is the diversity of how wildfire exposure is conceptualized and measured across literature. Broadly, three main exposure metrics are used: burned area, smoke exposure, and fire occurrence or detection. Burned areas, typically derived from satellite imagery or official fire records, is widely employed as a proxy for wildfire severity and spatial extent. While it captures the ecological scale of events, it does not necessarily correspond to human exposure levels. Smoke exposure, by contrast, is most directly linked to population‐level health impacts and is commonly estimated through ground‐based air quality measurements (e.g., PM_2_._5_), satellite‐derived aerosol optical depth, or chemical transport models. A third category, fire occurrence or detection, captures hotspot counts, ignition events, or binary presence indicators and is useful for identifying exposure timing but offers limited detail on intensity or health‐relevant pollutant load.

Furthermore, the accessibility and resolution of exposure data significantly shape research design and publication patterns. In regions like North America and Australia, satellite‐based wildfire detection and smoke plume products (e.g., NOAA's Hazard Mapping System or NASA's MODIS and VIIRS) are widely available and straightforward to use, enabling more frequent and sophisticated analyses. By contrast, limited availability of comparable global products, particularly those focused on smoke plumes, can constrain wildfire‐health research elsewhere, contributing to geographic disparities in the literature. Similarly, the type and availability of health data—ranging from administrative and cohort data to surveillance systems—can profoundly influence study feasibility and scope. The scarcity of detailed health data sets in many regions underscores a key barrier to advancing wildfire‐health research globally.

**Figure 13:**
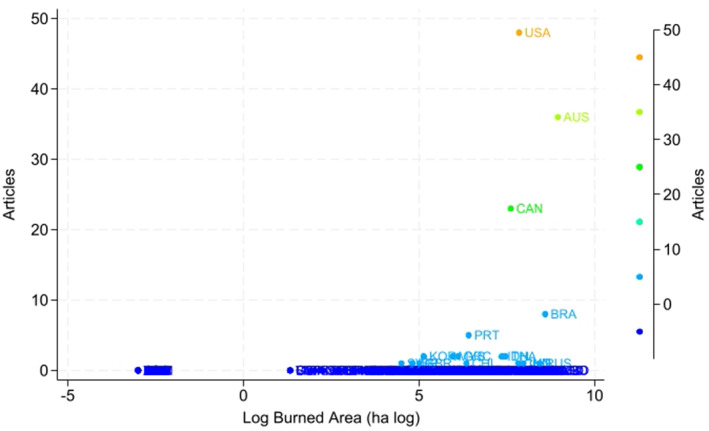
Relationship between wildfire severity and research attention across countries from 2000 to 2023.

Severity is represented by the logarithm of total burned area (hectares) in each country, plotted against the number of published articles on wildfire and public health. Countries such as the United States, Australia, and Canada show both extensive burned areas and high publication output, suggesting that severe and impactful wildfires in these regions have spurred greater scientific attention. In contrast, countries with comparable or large burned areas but limited research output indicate potential gaps in global wildfire‐health scholarship. Figure 13 reveals a significant mismatch between wildfire severity—measured by the logarithm of total burned area and the number of articles published across different countries. A positive correlation is evident in some cases: for example, the United States and Australia experience extensive burned areas and also produce high volumes of scholarly research, suggesting more severe wildfire events tends to spur greater scientific attention. However, this correlation does not consistently apply across all countries. Brazil, despite experiencing substantial wildfire areas, does notexhibt a comparable research output. Similarly, several countries with significant wildfire activity have little to no academic or journalistic publications, highlighting how research priorities do not always align with fire severity or potential community impacts. Future work should therefore emphasize expanding access to high‐quality exposure and health data and promoting the use of globally available satellite products to reduce geographic bias in wildfire‐health scholarship.

Recognizing these distinctions is crucial for interpreting the findings of wildfire–health studies and comparing results across regions and time periods. Studies focusing solely on burned area may underestimate population exposure, while those measuring only smoke may overlook ecological severity or spatial distribution. Integrating multiple exposure metrics within a single study would enhance the comprehensiveness of wildfire impact assessments and provide a more nuanced understanding of how different exposure dimensions interact with public health outcomes. Moreover, insights from this review point to broader methodological and thematic gaps that extend beyond exposure measurement alone. Mental health impacts, for example, remain significantly underexplored relative to physical health outcomes, and research seldom disaggregates results by demographic subgroups such as children, older adults, and socially marginalized populations. Addressing these gaps will require more integrated and equity‐focused research designs that combine diverse exposure metrics, broaden the scope of health outcomes examined, and expand data availability in underrepresented regions. As wildfire activity intensifies under climate change, future scholarship must prioritize harmonizing exposure definitions, incorporating psychosocial and demographic dimensions, and developing composite indicators that better capture the multifaceted nature of wildfire impacts on human health.

### 4.4 Unveiling the Shadows: The Need for Demographic‐Specific Research in Wildfire Health Impacts

The intricate dance between wildfires and human health does not follow a universal rhythm; instead, it varies significantly across the spectrum of socioeconomic and cultural backgrounds. This variation underscores the imperative for a more nuanced approach to understanding and mitigating the health impacts of wildfires, especially among the most vulnerable populations. The current landscape of wildfire health impact research, with its tendency toward aggregating data across the general population, risks oversimplifying the complex interplay of factors that influence health outcomes in the wake of such disasters. The interaction of socioeconomic status and cultural practices with wildfire exposure presents a multifaceted challenge. These factors not only determine the likelihood of experiencing wildfires but also shape the capacity of individuals and communities to respond effectively. For instance, the availability of resources like air filtration systems and healthcare services can significantly mitigate the adverse health effects of wildfires. However, access to these critical resources is often unevenly distributed, reflecting broader societal inequalities. Cultural factors, including traditional practices and community norms, further influence how warnings are received and the willingness to evacuate, which can have life‐or‐death consequences during wildfire events.

The prevailing focus on certain demographics, notably the elderly and children, highlights a recognition of their increased vulnerability due to physiological and dependency reasons. While this focus is undoubtedly warranted, it raises concerns about the potential neglect of other groups that also face significant risks but are less represented in research. The infrequent study of demographics such as minority communities or economically disadvantaged groups suggests a gap in our understanding of how wildfires affect these populations. Such gaps in research not only hinder the development of tailored interventions but also perpetuate inequalities in disaster preparedness and response.

To bridge this gap, future research endeavours must prioritize inclusivity, targeting a broader array of demographic groups to capture the full diversity of wildfire health impacts. This shift towards a more granular research approach would facilitate the development of targeted public health interventions and emergency services, ensuring that the unique needs of all vulnerable groups are met. Moreover, it would enable policymakers to craft regulations and policies that are informed by a comprehensive understanding of the disparities in wildfire exposure and susceptibility. The frequency with which certain demographics appear in research not only reflects current priorities but also influences future policy directions. Populations more frequently represented in research are likely to see their needs more directly addressed in policy and intervention designs. Conversely, the underrepresentation of specific groups can lead to a critical oversight, leaving them less protected against the ravages of wildfires. It is imperative for both researchers and policymakers to critically examine these trends, actively working to counteract biases and ensure that no group is left behind in the collective effort to mitigate the health impacts of wildfires.

### 4.5 Embracing Innovation: The Role of Machine Learning in Wildfire Health Impact Research

The evolving landscape of wildfire health impact research is marked by a gradual but significant shift toward more sophisticated analytical methodologies. Among these, machine learning emerges as a promising frontier, offering new avenues for understanding and addressing the complex interplay between wildfire incidents and public health outcomes. This shift, as documented in recent studies (Jain et al., 2020; Reid et al., 2015; Watson et al., 2019), underscores the potential of machine learning to revolutionize the way researchers approach the data‐intensive challenges inherent in this field.

While traditional statistical methods, such as linear regression, have long dominated the landscape of health impact analysis, the advent of machine learning techniques has introduced a paradigm shift. The initial utilization of machine learning in wildfire health research dates back to the early 2000s, but its widespread adoption has notably accelerated post‐2021. This trend reflects a growing recognition of the limitations of conventional methods in capturing the multifaceted effects of wildfires on health and the environment. Machine learning, with its ability to handle large data sets and uncover complex patterns, offers a robust tool for enhancing our understanding of wildfire‐related health risks. Studies by Zou et al. (2019) highlight the application of machine learning‐based data fusion algorithms for estimating surface PM2.5 concentrations, a key metric for assessing the health impacts of wildfire smoke. Such advancements in predictive accuracy are crucial for developing effective early warning systems and preparing healthcare responses to mitigate the adverse effects of smoke exposure.

The potential applications of machine learning in this domain extend far beyond exposure assessment. By analyzing vast amounts of data from various sources, machine learning algorithms can identify previously unrecognized patterns and trends in wildfire occurrences and their health implications. This capability not only enhances our understanding of the direct and indirect effects of wildfires but also informs the development of targeted public health interventions. Furthermore, machine learning can play a pivotal role in fire prevention and control strategies, offering innovative solutions based on predictive analytics and risk modeling. Given the promising results from early adopters of machine learning in wildfire health impact research, there is a compelling case for broader adoption of these techniques. Researchers are encouraged to explore the potential of machine learning, not as a replacement for traditional statistical methods, but as a complementary tool that can provide deeper insights and more accurate predictions. The integration of machine learning into research methodologies requires a multidisciplinary approach, combining expertise in data science, public health, and environmental science.

## 5 Conclusion

The comprehensive review on the health impacts of wildfires underscores the intricate and multifaceted relationship between these natural disasters and public health. As wildfire continues to surge in frequency and intensity, driven by climate change and altered fire regimes, their effects on human health extend well beyond the immediate risks. This review highlights the significant physical, psychological, and social dimensions of health consequences associated with wildfires. Respiratory and cardiovascular issues, prolonged exposure to poor air quality, population displacement, and psychological trauma are some of the critical health outcomes identified. The synthesis of diverse studies emphasizes the need for a more holistic understanding of these impacts to inform effective public health strategies and interventions.

One of the key insights from this review is the uneven distribution of research focus across different populations and study designs. While general populations and adults receive considerable attention, vulnerable groups such as children, the elderly, and the youth are underrepresented. This gap in research highlights the necessity for a more inclusive approach that addresses the unique vulnerabilities and needs of these groups. Additionally, the predominance of observational study designs over experimental ones reflects the context‐specific constraints and objectives of wildfire health research. However, there is a growing need for innovative methodologies, such as machine learning, to enhance the precision and depth of analysis in this field.

As we move forward, it is crucial to prioritize comprehensive and inclusive research efforts that capture the full spectrum of health impacts of wildfires. Advances in research methodologies, coupled with a focus on vulnerable populations, will enable the development of targeted public health policies and interventions. Bridging the gaps in current research and embracing innovative analytical tools will contribute to a more resilient and informed response to the escalating challenge of wildfires. By fostering a deeper understanding of the health ramifications of wildfires, we can better prepare for and mitigate the impacts of these increasingly prevalent natural disasters, ensuring a more robust public health framework for the future.

## Conflict of Interest

The authors declare no conflicts of interest relevant to this study.

## Data Availability

No new data was generated in this study. All data analyzed in this review are derived from previously published articles, which are cited throughout the manuscript and available through their respective journals and databases. Literature screening and extraction were conducted using the Web of Science Core Collection (Clarivate Analytics), and all cited works can be accessed via institutional subscriptions or publicly available journal platforms. No custom software was developed for this study. Bibliographic screening and management were performed using standard reference management tools (Zotero), and figure production was conducted using Microsoft Excel and StataSE (version 18.0).

## References

[gh270107-bib-0001] Abdo, M. , Ward, I. , O'Dell, K. , Ford, B. , Pierce, J. R. , Fischer, E. V. , & Crooks, J. L. (2019). Impact of wildfire smoke on adverse pregnancy outcomes in Colorado, 2007–2015. International Journal of Environmental Research and Public Health, 16(19). Article 19. 10.3390/ijerph16193720 PMC6801422 31581673

[gh270107-bib-0002] Adetona, O. , Reinhardt, T. E. , Domitrovich, J. , Broyles, G. , Adetona, A. M. , Kleinman, M. T. , et al. (2016). Review of the health effects of wildland fire smoke on wildland firefighters and the public. Inhalation Toxicology, 28(3), 95–139. 10.3109/08958378.2016.1145771 26915822

[gh270107-bib-0003] Archibald, S. (2016). Managing the human component of fire regimes: Lessons from Africa. Philosophical Transactions of the Royal Society B: Biological Sciences, 371(1696), 20150346. 10.1098/rstb.2015.0346 PMC4874421 27216516

[gh270107-bib-0004] Artés, T. , Oom, D. , De Rigo, D. , Durrant, T. H. , Maianti, P. , Libertà, G. , & San‐Miguel‐Ayanz, J. (2019). A global wildfire dataset for the analysis of fire regimes and fire behaviour. Scientific Data, 6(1), 296. 10.1038/s41597-019-0312-2 31784525 PMC6884633

[gh270107-bib-0005] Barbosa, J. V. , Nunes, R. A. O. , Alvim‐Ferraz, M. C. M. , Martins, F. G. , & Sousa, S. I. V. (2022). Health and economic burden of the 2017 Portuguese extreme wildland fires on children. IJERPH, 19(1), 1–12. 10.3390/ijerph19010593 PMC8745015 35010865

[gh270107-bib-0006] Barbosa, J. V. , Nunes, R. A. O. , Alvim‐Ferraz, M. C. M. , Martins, F. G. , & Sousa, S. I. V. (2024). Health and economic burden of wildland fires PM_2.5_‐related pollution in Portugal—A longitudinal study. Environmental Research, 240, 117490. 10.1016/j.envres.2023.117490 37879391

[gh270107-bib-0007] Belleville, G. , Ouellet, M.‐C. , Lebel, J. , Ghosh, S. , Morin, C. M. , Bouchard, S. , et al. (2021). Psychological symptoms among evacuees from the 2016 Fort McMurray wildfires: A population‐based survey one year later. Frontiers in Public Health, 9, 655357. 10.3389/fpubh.2021.655357 34017813 PMC8130827

[gh270107-bib-0008] Beyene, T. , Murphy, V. E. , Gibson, P. G. , McDonald, V. M. , Van Buskirk, J. , Holliday, E. G. , et al. (2022). The impact of prolonged landscape fire smoke exposure on women with asthma in Australia. BMC Pregnancy and Childbirth, 22(1), 919. 10.1186/s12884-022-05231-8 36482359 PMC9733231

[gh270107-bib-0009] Bowman, D. M. J. S. , Williamson, G. J. , Abatzoglou, J. T. , Kolden, C. A. , Cochrane, M. A. , & Smith, A. M. S. (2017). Human exposure and sensitivity to globally extreme wildfire events. Nature Ecology & Evolution, 1(3), 0058. 10.1038/s41559-016-0058 28812737

[gh270107-bib-0010] Brown, M. R. G. , Agyapong, V. , Greenshaw, A. J. , Cribben, I. , Brett‐MacLean, P. , Drolet, J. , et al. (2019a). Significant PTSD and other mental health effects present 18 months after the Fort Mcmurray wildfire: Findings from 3,070 grades 7–12 students. Frontiers in Psychiatry, 10, 623. 10.3389/fpsyt.2019.00623 31543839 PMC6728415

[gh270107-bib-0011] Brown, M. R. G. , Agyapong, V. , Greenshaw, A. J. , Cribben, I. , Brett‐MacLean, P. , Drolet, J. , et al. (2019b). After the Fort McMurray wildfire there are significant increases in mental health symptoms in grade 7–12 students compared to controls. BMC Psychiatry, 19(1), 18. 10.1186/s12888-018-2007-1 30630501 PMC6329184

[gh270107-bib-0012] Brown, M. R. G. , Pazderka, H. , Agyapong, V. I. O. , Greenshaw, A. J. , Cribben, I. , Brett‐MacLean, P. , et al. (2021). Mental health symptoms unexpectedly increased in students aged 11–19 years during the 3.5 years after the 2016 Fort McMurray wildfire: Findings from 9,376 survey responses. Frontiers in Psychiatry, 12, 676256. 10.3389/fpsyt.2021.676256 34093284 PMC8172807

[gh270107-bib-0013] Bryant, R. A. , Gallagher, H. C. , Gibbs, L. , Pattison, P. , MacDougall, C. , Harms, L. , et al. (2017). Mental health and social networks after disaster. American Journal of Psychiatry, 174(3), 277–285. 10.1176/appi.ajp.2016.15111403 27838935

[gh270107-bib-0014] Bryant, R. A. , Gibbs, L. , Colin Gallagher, H. , Pattison, P. , Lusher, D. , MacDougall, C. , et al. (2021). The dynamic course of psychological outcomes following the Victorian Black Saturday bushfires. Australian and New Zealand Journal of Psychiatry, 55(7), 666–677. 10.1177/0004867420969815 33176436

[gh270107-bib-0015] Bryant, R. A. , Gibbs, L. , Gallagher, H. C. , Pattison, P. , Lusher, D. , MacDougall, C. , et al. (2018). Longitudinal study of changing psychological outcomes following the Victorian Black Saturday bushfires. Australian and New Zealand Journal of Psychiatry, 52(6), 542–551. 10.1177/0004867417714337 28605987

[gh270107-bib-0016] Bryant, R. A. , Waters, E. , Gibbs, L. , Gallagher, H. C. , Pattison, P. , Lusher, D. , et al. (2014). Psychological outcomes following the Victorian Black Saturday bushfires. Australian and New Zealand Journal of Psychiatry, 48(7), 634–643. 10.1177/0004867414534476 24852323

[gh270107-bib-0017] Casey, J. A. , Kioumourtzoglou, M.‐A. , Elser, H. , Walker, D. , Taylor, S. , Adams, S. , et al. (2021). Wildfire particulate matter in Shasta County, California and respiratory and circulatory disease‐related emergency department visits and mortality, 2013–2018. Environmental Epidemiology, 5(1), e124. 10.1097/EE9.0000000000000124 33778357 PMC7939433

[gh270107-bib-0018] Cavanagh, A. , Wilson, C. J. , Kavanagh, D. J. , & Caputi, P. (2018). Men and women's psychological outcomes in communities affected by bushfires. The Australian Community Psychologist, 29(2).

[gh270107-bib-0019] Ciciretti, R. , Barraza, F. , De la Barrera, F. , Urquieta, L. , & Cortes, S. (2022). Relationship between wildfire smoke and children's respiratory health in the metropolitan cities of central‐Chile. Atmosphere, 13(1), 58. Article 1. 10.3390/atmos13010058

[gh270107-bib-0020] Cleland, S. E. , Serre, M. L. , Rappold, A. G. , & West, J. J. (2021). Estimating the acute health impacts of fire‐originated PM_2.5_ exposure during the 2017 California wildfires: Sensitivity to choices of inputs. GeoHealth, 5(7), e2021GH000414. 10.1029/2021GH000414 PMC8247531 34250370

[gh270107-bib-0021] Crabbe, H. (2012). Risk of respiratory and cardiovascular hospitalisation with exposure to bushfire particulates: New evidence from Darwin, Australia. Environmental Geochemistry and Health, 34(6), 697–709. 10.1007/s10653-012-9489-4 23053929

[gh270107-bib-0022] DeFlorio‐Barker, S. , Crooks, J. , Reyes, J. , & Rappold, A. G. (2019). Cardiopulmonary effects of fine particulate matter exposure among older adults, during wildfire and non‐wildfire periods, in the United States 2008–2010. Environmental Health Perspectives, 127(3), 037006. 10.1289/EHP3860 30875246 PMC6768318

[gh270107-bib-0023] Dittrich, R. , & McCallum, S. (2020). How to measure the economic health cost of wildfires–A systematic review of the literature for northern America. International Journal of Wildland Fire, 29(11), 961–973. 10.1071/wf19091

[gh270107-bib-0024] Elliott, C. T. , Henderson, S. B. , & Wan, V. (2013). Time series analysis of fine particulate matter and asthma reliever dispensations in populations affected by forest fires. Environmental Health: A Global Access Science Source, 12(1), 11. 10.1186/1476-069X-12-11 23356966 PMC3582455

[gh270107-bib-0030] European Commission, Joint Research Centre (JRC) . (2024). Global Monthly Burned Area [2002–2023] [Dataset]. Global Wildfire Information System (GWIS). Retrieved from https://gwis.jrc.ec.europa.eu/apps/country.profile/downloads

[gh270107-bib-0025] Foo, D. , Heo, S. , Stewart, R. , Dhamrait, G. , Choi, H. M. , Song, Y. , & Bell, M. L. (2023). Wildfire smoke exposure during pregnancy and perinatal, obstetric, and early childhood health outcomes: A systematic review and meta‐analysis. Environmental Research, 241, 117527. 10.1016/j.envres.2023.117527 37931734

[gh270107-bib-0026] Ford, N. D. , Patel, S. A. , & Narayan, K. M. V. (2017). Obesity in low‐ and middle‐income countries: Burden, drivers, and emerging challenges. Annual Review of Public Health, 38(1), 145–164. 10.1146/annurev-publhealth-031816-044604 28068485

[gh270107-bib-0027] Friesinger, J. G. , Topor, A. , Bøe, T. D. , & Larsen, I. B. (2019). Studies regarding supported housing and the built environment for people with mental health problems: A mixed‐methods literature review. Health & Place, 57, 44–53. 10.1016/j.healthplace.2019.03.006 30959400

[gh270107-bib-0028] Gallagher, H. C. , Block, K. , Gibbs, L. , Forbes, D. , Lusher, D. , Molyneaux, R. , et al. (2019). The effect of group involvement on post‐disaster mental health: A longitudinal multilevel analysis. Social Science & Medicine, 220, 167–175. 10.1016/j.socscimed.2018.11.006 30447481

[gh270107-bib-0029] Gao, Y. , Huang, W. , Yu, P. , Xu, R. , Yang, Z. , Gasevic, D. , et al. (2023). Long‐term impacts of non‐occupational wildfire exposure on human health: A systematic review. Environmental Pollution, 320, 121041. 10.1016/j.envpol.2023.121041 36639044

[gh270107-bib-0031] Gould, K. , & Lewis, T. (2016). Green gentrification: Urban sustainability and the struggle for environmental justice. Routledge. 10.4324/9781315687322

[gh270107-bib-0032] Haikerwal, A. , Akram, M. , Del Monaco, A. , Smith, K. , Sim, M. R. , Meyer, M. , et al. (2015). Impact of fine particulate matter (PM_2.5_) exposure during wildfires on cardiovascular health outcomes. Journal of the American Heart Association, 4(7), e001653. 10.1161/JAHA.114.001653 26178402 PMC4608063

[gh270107-bib-0033] Hanigan, I. C. , Johnston, F. H. , & Morgan, G. G. (2008). Vegetation fire smoke, indigenous status and cardio‐respiratory hospital admissions in Darwin, Australia, 1996–2005: A time‐series study. Environmental Health, 7(1), 42. 10.1186/1476-069X-7-42 18680605 PMC2535774

[gh270107-bib-0034] Heaney, A. , Stowell, J. D. , Liu, J. C. , Basu, R. , Marlier, M. , & Kinney, P. (2022). Impacts of fine particulate matter from wildfire smoke on respiratory and cardiovascular health in California. GeoHealth, 6(6), e2021GH000578. 10.1029/2021GH000578 PMC9166629 35795228

[gh270107-bib-0035] Heft‐Neal, S. , Driscoll, A. , Yang, W. , Shaw, G. , & Burke, M. (2022). Associations between wildfire smoke exposure during pregnancy and risk of preterm birth in California. Environmental Research, 203, 111872. 10.1016/j.envres.2021.111872 34403668

[gh270107-bib-0036] Henderson, S. B. , Brauer, M. , Macnab, Y. C. , & Kennedy, S. M. (2011). Three measures of forest fire smoke exposure and their associations with respiratory and cardiovascular health outcomes in a population‐based cohort. Environmental Health Perspectives, 119(9), 1266–1271. 10.1289/ehp.1002288 21659039 PMC3230386

[gh270107-bib-0037] Henry, S. , Ospina, M. B. , Dennett, L. , & Hicks, A. (2021). Assessing the risk of respiratory‐related healthcare visits associated with wildfire smoke exposure in children 0–18 years old: A systematic review. International Journal of Environmental Research and Public Health, 18(16), 8799. 10.3390/ijerph18168799 34444546 PMC8392577

[gh270107-bib-0038] Holstius, D. M. , Reid, C. E. , Jesdale, B. M. , & Morello‐Frosch, R. (2012). Birth weight following pregnancy during the 2003 Southern California Wildfires. Environmental Health Perspectives, 120(9), 1340–1345. 10.1289/ehp.1104515 22645279 PMC3440113

[gh270107-bib-0039] Hong, J. S. , Hyun, S. Y. , Lee, J. H. , & Sim, M. (2022). Mental health effects of the Gangwon wildfires. BMC Public Health, 22(1), 1183. 10.1186/s12889-022-13560-8 35701801 PMC9195206

[gh270107-bib-0040] Hutchinson, J. A. , Vargo, J. , Milet, M. , French, N. H. F. , Billmire, M. , Johnson, J. , & Hoshiko, S. (2018). The San Diego 2007 wildfires and Medi‐Cal emergency department presentations, inpatient hospitalizations, and outpatient visits: An observational study of smoke exposure periods and a bidirectional case‐crossover analysis. PLoS Medicine, 15(7), e1002601. 10.1371/journal.pmed.1002601 29990362 PMC6038982

[gh270107-bib-0041] Jain, P. , Coogan, S. C. , Subramanian, S. G. , Crowley, M. , Taylor, S. , & Flannigan, M. D. (2020). A review of machine learning applications in wildfire science and management. Environmental Reviews, 28(4), 478–505. 10.1139/er-2020-0019

[gh270107-bib-0042] Jiao, A. , Headon, K. , Han, T. , Umer, W. , & Wu, J. (2023). Associations between short‐term exposure to wildfire particulate matter and respiratory outcomes: A systematic review. Science of the Total Environment, 907, 168134. 10.1016/j.scitotenv.2023.168134 39491190

[gh270107-bib-0043] Johnston, F. H. , Henderson, S. B. , Chen, Y. , Randerson, J. T. , Marlier, M. , Defries, R. S. , et al. (2012). Estimated global mortality attributable to smoke from landscape fires. Environmental Health Perspectives, 120(5), 695–701. 10.1289/ehp.1104422 22456494 PMC3346787

[gh270107-bib-0044] Kondo, M. C. , Fluehr, J. M. , McKeon, T. , & Branas, C. C. (2018). Urban green space and its impact on human health. International Journal of Environmental Research and Public Health, 15(3), 445. 10.3390/ijerph15030445 29510520 PMC5876990

[gh270107-bib-0045] Korsiak, J. , Pinault, L. , Christidis, T. , Burnett, R. T. , Abrahamowicz, M. , & Weichenthal, S. (2022). Long‐term exposure to wildfires and cancer incidence in Canada: A population‐based observational cohort study. The Lancet Planetary Health, 6(5), e400–e409. 10.1016/S2542-5196(22)00067-5 35550079

[gh270107-bib-0046] Künzli, N. , Avol, E. , Wu, J. , Gauderman, W. J. , Rappaport, E. , Millstein, J. , et al. (2006). Health effects of the 2003 Southern California wildfires on children. American Journal of Respiratory and Critical Care Medicine, 174(11), 1221–1228. 10.1164/rccm.200604-519OC 16946126 PMC2648104

[gh270107-bib-0047] Le, G. E. , Breysse, P. N. , McDermott, A. , Eftim, S. E. , Geyh, A. , Berman, J. D. , & Curriero, F. C. (2014). Canadian forest fires and the effects of long‐range transboundary air pollution on hospitalizations among the elderly. ISPRS International Journal of Geo‐Information, 3(2), 713–731. Article 2. 10.3390/ijgi3020713 36405525 PMC9673582

[gh270107-bib-0048] Liu, G. , Zhang, L. , He, B. , Jin, X. , Zhang, Q. , Razafindrabe, B. , & You, H. (2015). Temporal changes in extreme high temperature, heat waves and relevant disasters in Nanjing metropolitan region, China. Natural Hazards, 76(2), 1415–1430. 10.1007/s11069-014-1556-y

[gh270107-bib-0049] Liu, J. C. , Wilson, A. , Mickley, L. J. , Dominici, F. , Ebisu, K. , Wang, Y. , et al. (2017). Wildfire‐specific fine particulate matter and risk of hospital admissions in urban and rural counties. Epidemiology, 28(1), 77–85. 10.1097/ede.0000000000000556 27648592 PMC5130603

[gh270107-bib-0050] Liu, J. C. , Wilson, A. , Mickley, L. J. , Ebisu, K. , Sulprizio, M. P. , Wang, Y. , et al. (2017). Who among the elderly is most vulnerable to exposure to and health risks of fine particulate matter from wildfire smoke? American Journal of Epidemiology, 186(6), 730–735. 10.1093/aje/kwx141 28525551 PMC5860049

[gh270107-bib-0051] Magzamen, S. , Gan, R. W. , Liu, J. , O'Dell, K. , Ford, B. , Berg, K. , et al. (2021). Differential cardiopulmonary health impacts of local and long‐range transport of wildfire smoke. GeoHealth, 5(3), e2020GH000330. 10.1029/2020GH000330 PMC8900982 35281479

[gh270107-bib-0052] Mahsin, M. D. , Cabaj, J. , & Saini, V. (2022). Respiratory and cardiovascular condition‐related physician visits associated with wildfire smoke exposure in Calgary, Canada, in 2015: A population‐based study. International Journal of Epidemiology, 51(1), 166–178. 10.1093/ije/dyab206 34561694

[gh270107-bib-0053] Mao, W. , Adu, M. , Eboreime, E. , Shalaby, R. , Nkire, N. , Agyapong, B. , et al. (2022). Post‐traumatic stress disorder, major depressive disorder, and wildfires: A fifth‐year postdisaster evaluation among residents of Fort McMurray. International Journal of Environmental Research and Public Health, 19(15), 9759. 10.3390/ijerph19159759 35955114 PMC9368448

[gh270107-bib-0054] Moher, D. , Liberati, A. , Tetzlaff, J. , Altman, D. G. , & PRISMA Group . (2009). Preferred reporting items for systematic reviews and meta‐analyses: The PRISMA statement. PLoS Medicine, 6(7), e1000097. 10.1371/journal.pmed.1000097 19621072 PMC2707599

[gh270107-bib-0055] Moitra, S. , Tabrizi, A. F. , Fathy, D. , Kamravaei, S. , Miandashti, N. , Henderson, L. , et al. (2021). Short‐term acute exposure to wildfire smoke and lung function among royal Canadian mounted police (RCMP) officers. International Journal of Environmental Research and Public Health, 18(22), 11787. 10.3390/ijerph182211787 34831540 PMC8618710

[gh270107-bib-0056] Moosavi, S. , Nwaka, B. , Akinjise, I. , Corbett, S. E. , Chue, P. , Greenshaw, A. J. , et al. (2019). Mental health effects in primary care patients 18 months after a major wildfire in Fort McMurray: Risk increased by social demographic issues, clinical antecedents, and degree of fire exposure. Frontiers in Psychiatry, 10, 683. 10.3389/fpsyt.2019.00683 31620033 PMC6760025

[gh270107-bib-0057] Morgan, G. , Sheppeard, V. , Khalaj, B. , Ayyar, A. , Lincoln, D. , Jalaludin, B. , et al. (2010). Effects of bushfire smoke on daily mortality and hospital admissions in Sydney, Australia. Epidemiology, 21(1), 47–55. 10.1097/EDE.0b013e3181c15d5a 19907335

[gh270107-bib-0058] Murphy, V. E. , Jensen, M. E. , Holliday, E. G. , Giles, W. B. , Barrett, H. L. , Callaway, L. K. , et al. (2022). Effect of asthma management with exhaled nitric oxide versus usual care on perinatal outcomes. European Respiratory Journal, 60(5), 2200298. 10.1183/13993003.00298-2022 35777773 PMC9669403

[gh270107-bib-0059] Ontawong, A. , Saokaew, S. , Jamroendararasame, B. , & Duangjai, A. (2020). Impact of long‐term exposure wildfire smog on respiratory health outcomes. Expert Review of Respiratory Medicine, 14(5), 527–531. 10.1080/17476348.2020.1740089 32156169

[gh270107-bib-0060] Orr, A. , Migliaccio, A. L. , Buford, M. , Ballou, S. , & Migliaccio, C. T. (2020). Sustained effects on lung function in community members following exposure to hazardous PM_2.5_ levels from wildfire smoke. Toxics, 8(3), 53. 10.3390/toxics8030053 32764367 PMC7560437

[gh270107-bib-0061] Park, B. Y. , Boles, I. , Monavvari, S. , Patel, S. , Alvarez, A. , Phan, M. , et al. (2022). The association between wildfire exposure in pregnancy and foetal gastroschisis: A population‐based cohort study. Paediatric & Perinatal Epidemiology, 36(1), 45–53. 10.1111/ppe.12823 34797578

[gh270107-bib-0062] Parslow, R. A. , Jorm, A. F. , & Christensen, H. (2006). Associations of pre‐trauma attributes and trauma exposure with screening positive for PTSD: Analysis of a community‐based study of 2085 young adults. Psychological Medicine, 36(3), 387–395. 10.1017/S0033291705006306 16255836

[gh270107-bib-0063] Rangel, M. A. , & Vogl, T. S. (2019). Agricultural fires and health at birth. The Review of Economics and Statistics, 101(4), 616–630. 10.1162/rest_a_00806

[gh270107-bib-0064] Reid, C. E. , Jerrett, M. , Petersen, M. L. , Pfister, G. G. , Morefield, P. E. , Tager, I. B. , et al. (2015). Spatiotemporal prediction of fine particulate matter during the 2008 northern California wildfires using machine learning. Environmental Science & Technology, 49(6), 3887–3896. 10.1021/es505846r 25648639

[gh270107-bib-0065] Requia, W. J. , Amini, H. , Adams, M. D. , & Schwartz, J. D. (2022). Birth weight following pregnancy wildfire smoke exposure in more than 1.5 million newborns in Brazil: A nationwide case‐control study. Lancet Regional Health. Americas, 11, 100229. 10.1016/j.lana.2022.100229 36778934 PMC9903686

[gh270107-bib-0066] Requia, W. J. , Kill, E. , Papatheodorou, S. , Koutrakis, P. , & Schwartz, J. D. (2022). Prenatal exposure to wildfire‐related air pollution and birth defects in Brazil. Journal of Exposure Science and Environmental Epidemiology, 32(4), 596–603. 10.1038/s41370-021-00380-y 34504295

[gh270107-bib-0067] Ritchie, A. , Sautner, B. , Omege, J. , Denga, E. , Nwaka, B. , Akinjise, I. , et al. (2021). Long‐term mental health effects of a devastating wildfire are amplified by sociodemographic and clinical antecedents in college students. Disaster Medicine and Public Health Preparedness, 15(6), 707–717. 10.1017/dmp.2020.87 32536354

[gh270107-bib-0068] Rodney, R. M. , Swaminathan, A. , Calear, A. L. , Christensen, B. K. , Lal, A. , Lane, J. , et al. (2021). Physical and mental health effects of bushfire and smoke in the Australian capital territory 2019–20. Frontiers in Public Health, 9, 682402. 10.3389/fpubh.2021.682402 34722432 PMC8551801

[gh270107-bib-0069] Santos, M. Y. , Leite, V. , Carvalheira, A. , de Araújo, A. T. , & Cruz, J. (2015). Are wildfires and pneumonia spatially and temporally related? In F. Ortuño & I. Rojas (Eds.), Bioinformatics and Biomedical Engineering (pp. 42–53). Springer International Publishing. 10.1007/978-3-319-16483-0_5

[gh270107-bib-0070] Schöllnberger, H. , Aden, J. , & Scott, B. R. (2002). Respiratory tract deposition efficiencies: Evaluation of effects from smoke released in the Cerro Grande forest fire. Journal of Aerosol Medicine, 15(4), 387–399. 10.1089/08942680260473461 12581505

[gh270107-bib-0071] Search Results | National Interagency Fire Center . (2025). Retrieved from https://www.nifc.gov/search

[gh270107-bib-0072] Silveira, S. , Kornbluh, M. , Withers, M. C. , Grennan, G. , Ramanathan, V. , & Mishra, J. (2021). Chronic mental health sequelae of climate change extremes: A case study of the deadliest Californian wildfire. International Journal of Environmental Research and Public Health, 18(4), 1487. Article 4. 10.3390/ijerph18041487 33557397 PMC7915298

[gh270107-bib-0073] The human cost of disasters: An overview of the last 20 years (2000‐2019) | UNDRR . (2020). The human cost of disasters: An overview of the last 20 years (2000‐2019) | UNDRR. Retrieved from https://www.undrr.org/publication/human‐cost‐disasters‐overview‐last‐20‐years‐2000‐2019

[gh270107-bib-0074] To, P. , Eboreime, E. , & Agyapong, V. I. (2021). The impact of wildfires on mental health: A scoping review. Behavioral Sciences, 11(9), 126. 10.3390/bs11090126 34562964 PMC8466569

[gh270107-bib-0075] Usher, K. , Durkin, J. , Douglas, L. , Coffey, Y. , & Bhullar, N. (2022). Coping styles and mental health outcomes of community members affected by black summer 2019‐20 bushfires in Australia. International Journal of Mental Health Nursing, 31(5), 1176–1185. 10.1111/inm.13035 35731685

[gh270107-bib-0076] van der Werf, G. R. , Randerson, J. T. , Giglio, L. , van Leeuwen, T. T. , Chen, Y. , Rogers, B. M. , et al. (2017). Global fire emissions estimates during 1997–2016. Earth System Science Data, 9(2), 697–720. 10.5194/essd-9-697-2017

[gh270107-bib-0077] Verstraeten, B. S. E. , Elgbeili, G. , Hyde, A. , King, S. , & Olson, D. M. (2021). Maternal mental health after a wildfire: Effects of social support in the Fort McMurray wood buffalo study. The Canadian Journal of Psychiatry, 66(8), 710–718. 10.1177/0706743720970859 33172310 PMC8320544

[gh270107-bib-0078] Vizzuality . (2024). Central African Republic deforestation rates & statistics | GFW. Retrieved from https://www.globalforestwatch.org/dashboards/country/CAF?category=undefined

[gh270107-bib-0079] Watson, G. L. , Telesca, D. , Reid, C. E. , Pfister, G. G. , & Jerrett, M. (2019). Machine learning models accurately predict ozone exposure during wildfire events. Environmental Pollution, 254, 112792. 10.1016/j.envpol.2019.06.088 31421571

[gh270107-bib-0080] Wen, B. , Wu, Y. , Xu, R. , Guo, Y. , & Li, S. (2022). Excess emergency department visits for cardiovascular and respiratory diseases during the 2019–20 bushfire period in Australia: A two‐stage interrupted time‐series analysis. Science of the Total Environment, 809, 152226. 10.1016/j.scitotenv.2021.152226 34890657

[gh270107-bib-0081] Yao, J. , Brauer, M. , Wei, J. , McGrail, K. M. , Johnston, F. H. , & Henderson, S. B. (2020). Sub‐daily exposure to fine particulate matter and ambulance dispatches during wildfire seasons: A case‐crossover study in British Columbia, Canada. Environmental Health Perspectives, 128(6), 067006. 10.1289/EHP5792 32579089 PMC7313403

[gh270107-bib-0082] Yu, P. , Xu, R. , Li, S. , Yue, X. , Chen, G. , Ye, T. , et al. (2022). Exposure to wildfire‐related PM_2.5_ and site‐specific cancer mortality in Brazil from 2010 to 2016: A retrospective study. PLoS Medicine, 19(9), e1004103. 10.1371/journal.pmed.1004103 36121854 PMC9529133

[gh270107-bib-0083] Zhang, L. , Xu, H. , & Pan, J. (2023). Investigating the relationship between landscape design types and human thermal comfort: Case study of Beijing Olympic forest park. Sustainability, 15(4), 2969. 10.3390/su15042969

[gh270107-bib-0084] Zhang, S. , Zhang, X. , Niu, D. , Fang, Z. , Chang, H. , & Lin, Z. (2023). Physiological equivalent temperature‐based and universal thermal climate index‐based adaptive‐rational outdoor thermal comfort models. Building and Environment, 228, 109900. 10.1016/j.buildenv.2022.109900

[gh270107-bib-0085] Zhou, Y. , Kong, R. , Xu, Z. , Xu, L. , & Cheng, S. (2025). Comparative and interpretative analysis of CNN and transformer models in predicting wildfire spread using remote sensing data. Journal of Geophysical Research: Machine Learning and Computation, 2(2), e2024JH000409. 10.1029/2024JH000409

[gh270107-bib-0086] Zou, Y. , O'Neill, S. M. , Larkin, N. K. , Alvarado, E. C. , Solomon, R. , Mass, C. , et al. (2019). Machine learning‐based integration of high‐resolution wildfire smoke simulations and observations for regional health impact assessment. International Journal of Environmental Research and Public Health, 16(12). Article 12. 10.3390/ijerph16122137 PMC6617359 31212933

